# A novel highly thermostable xylanase stimulated by Ca^2+^ from *Thermotoga thermarum*: cloning, expression and characterization

**DOI:** 10.1186/1754-6834-6-26

**Published:** 2013-02-18

**Authors:** Hao Shi, Yu Zhang, Xun Li, Yingjuan Huang, Liangliang Wang, Ye Wang, Huaihai Ding, Fei Wang

**Affiliations:** 1 College of Chemical Engineering, Nanjing Forestry University, 210037, Nanjing, China; 2 Jiangsu Key Lab of Biomass-Based Green Fuels and Chemicals, 213337, Nanjing, China

**Keywords:** Xylanase, Xylan, Thermostability, Beechwood, Oat spelt, Birchwood

## Abstract

**Background:**

Xylanase is an important component of hemicellulase enzyme system. Since it plays an important role in the hydrolysis of hemicellulose into xylooligosaccharides (XOs), high thermostable xylanase has been the focus of much recent attention as powerful enzyme as well as in the field of biomass utilization.

**Results:**

A xylanase gene (*xyn10A*) with 3,474 bp was cloned from the extremely thermophilic bacterium *Thermotoga thermarum* that encodes a protein containing 1,158 amino acid residues. Based on amino acid sequence homology, hydrophobic cluster and three dimensional structure analyses, it was attested that the xylanase belongs to the glycoside hydrolase (GH) families 10 with five carbohydrate binding domains. When the xylanase gene was cloned and expressed in *Escherichia coli* BL21 (DE3), the specific enzyme activity of xylanase produced by the recombinant strain was up to 145.8 U mg^-1^. The xylanase was optimally active at 95°C, pH 7.0. In addition, it exhibited high thermostability over broad range of pH 4.0-8.5 and temperature 55-90°C upon the addition of 5 mM Ca^2+^. Confirmed by Ion Chromatography System (ICS) analysis, the end products of the hydrolysis of beechwood xylan were xylose, xylobiose, xylotriose, xylotetraose, xylopentaose and xylohexaose.

**Conclusions:**

The xylanase from *T*. *thermarum* is one of the hyperthermophilic xylanases that exhibits high thermostability, and thus, is a suitable candidate for generating XOs from cellulosic materials such as agricultural and forestry residues for the uses as prebiotics and precursors for further preparation of furfural and other chemicals.

## Background

Xylanases (EC 3.2.1.8) have played an important role in many industrial processes and have been applied as additives to enhance the quality of baked goods, animal feeds as well as to bleach kraft pulp [1]. The xylanases are also the crucial enzymes for the depolymerization of xylan by randomly hydrolyzing the β-1,4-glycosidic bonds to produce xylooligosaccharides with different lengths, thus they have great potential commercial value. Compared with other renewable resources, xylans, which are the main constituents of hemicellulose, are the world’s second most abundant resource after cellulose [2,3]. Xylan consists of α-1,4-linked β-D-xylopyranose units backbone and O-acetyl, α-L-arabinofuranosyl, α-1,2-linked glucuronic or 4-O-methylglucuronic acid side-chain substituent. The degree of polymerization in xylans and their side-chain substituents vary from the grasses (e.g. oat spelt), softwoods and hardwoods [4,5]. Due to the diversity in chemical structure of xylan derived from woods, cereal or other plant materials, it is widely known that to completely hydrolyze xylan it requires enzymes with different catalytic properties [6]. Among these hydrolytic enzymes, xylanases and xylosidases play the most important role in depolymerization of the xylan backbone, while other enzymes act on the side chain cleavage [7]. Based on the amino acid sequence homology, hydrophobic cluster, and three dimensional structural analysis, xylanases are classified mainly into two glycoside hydrolase (GH) families, 10 and 11, which enzyme activities are also present in GH 5, 7, 8, 16, 26, 43, 52 and 62 [2,4,8]. GH10 comprises enzymes with a number of known activities which are xylanase (EC 3.2.1.8), endo-1,3-beta-xylanase (EC 3.2.1.32) and cellobiohydrolase (EC:3.2.1.91).

Many new hyperthermophiles from bacteria and archaea have recently been isolated and the genomes of an increasing number have been sequenced [9-17], thus there are plenty of sequences can be obtained from the databases (http://www.ncbi.nlm.nih.gov/, http://www.cazy.org/). The genome sequence of *Thermotoga thermarum* was also reported last year (GenBank accession number: CP002351).

In this study, we described the cloning, expression and functional characterizations of Xyn10A, the xylanase from *Thermotoga thermarum* belonging to GH10.

## Results

### Cloning and sequence analysis of Xyn10A

A xylanase gene (Theth_1635), encoding 1,177 amino acids, based on the analysis of the genome sequence of *T*. *thermarum* DSM 5069, shares the highest amino acid sequence similarity of 51% with the annotated xylanases from *Thermotoga petrophila* RKU-1 (Genbank No. YP_001244458), *Thermotoga naphthophila* RKU-10 (Genbank No. YP_003346200) and *Thermotoga sp*. RQ2 (Genbank No. YP_001738920). Through BLAST of the catalytic domain (CD) of Xyn10A on GenBank, it shares the highest similarity of 78% with another xylanase in *T*. *thermarum* DSM 5069 (Genbank No. YP_004660782). There is a putative signal peptide sequence (20 amino acids, 60 bp) in Xyn10A when analyzed by SignalP 4.0 (http://www.cbs.dtu.dk/services/SignalP/), thus only the DNA fragment of *xyn10A* gene (3,474 bp) was amplified from the synthesized fragment with optimal codons in *E*. *coli*. The resulting gene was ligated to pET-20b at *NdeI* and *XhoI* sites to generate plasmid pET-20b-*xyn10A*.

### Expression and purification of recombinant xylanase Xyn10A

For functional analysis of the recombinant protein, the ORF region of the *xyn10A* gene was expressed in *E*. *coli* BL21 (DE3). The heterologous protein was over-expressed by inducing cells with 0.5 mM IPTG. The recombinant xylanase was purified through a heat treatment at 70°C for 30 min followed by a Ni-NTA affinity chromatography. The extracts from the bacteria harboring the construct Xyn10A exhibited a pure band at approximately 130 kDa by SDS-PAGE analysis (data not shown). Consistent with the theoretical molecular weight of the mature protein (130,949 Da) without the signal peptide, the target protein Xyn10A was successfully expressed and purified.

### Biochemical properties of the xylanase Xyn10A

**Figure 1:**
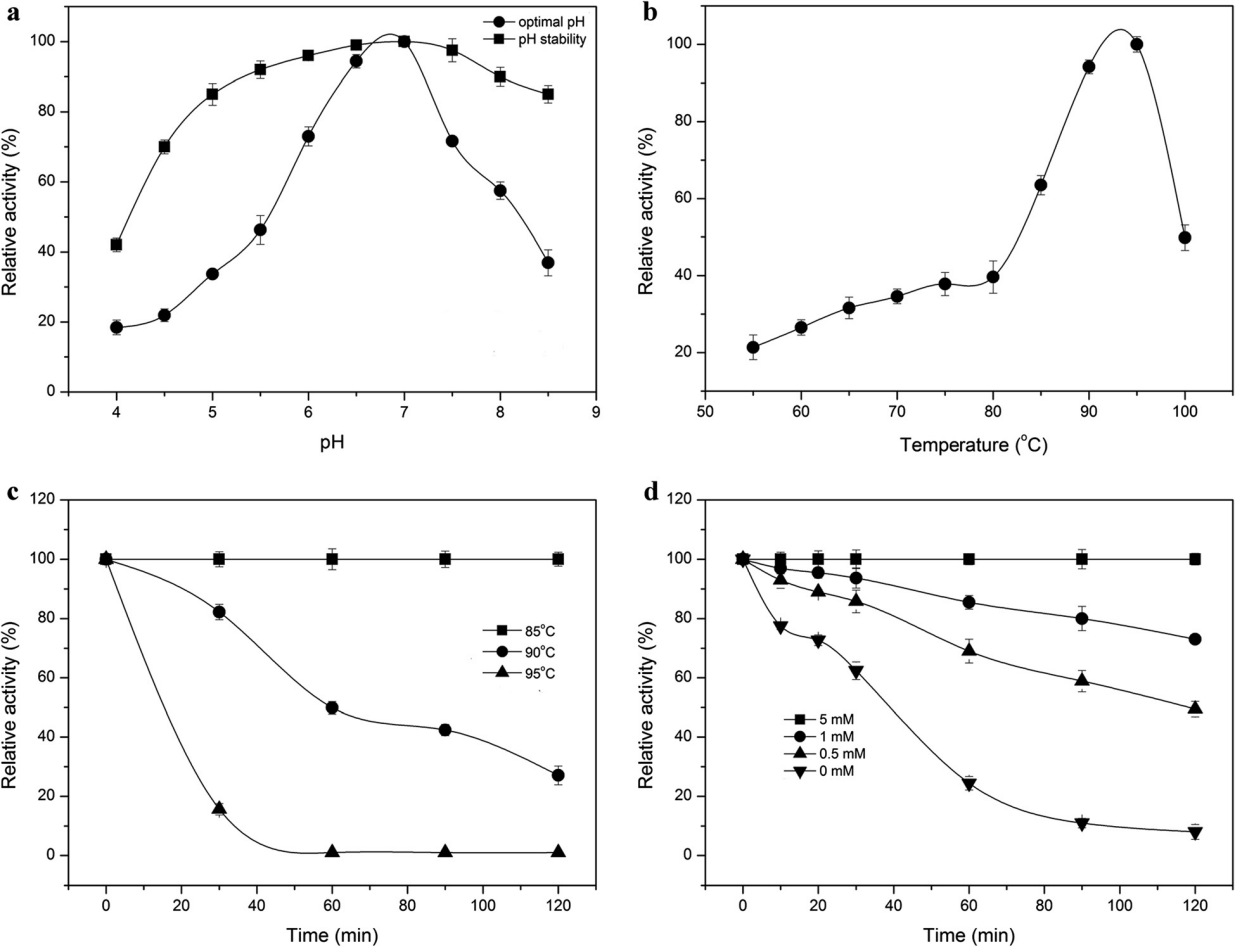
**Effects of pH and temperature on the activity and stability of the recombinant Xyn10A. a.** Optimal pH and pH stability of the Xyn10A. **b.** Effect of temperature on Xyn10A activity. **c.** The thermostability of the Xyn10A with 5 mM Ca^2+^. **d.** The thermostability of Xyn10A with different concentration of Ca^2+^. The residual activity was monitored, and the maximum activity was defined as 100% (a, b) or initial activity was defined as 100% (c, d). Values shown were the mean of the average of three experiments, and the variation about the mean was below 5%.

The Xyn10A displayed the highest enzyme activity at pH 7.0 (Figure 1a), while its relative activity all remained high, approximately 40% of the maximum activity, in the range of pH 5.5 to 8.5. It retained more than 60% of its activity at 60°C for 1 h when tested in the pH range of 4.5 to 8.5 (Figure 1a). The xylanase exhibited its optimal activity at 95°C (Figure 1b), and retained almost 100% of its initial activity when incubated at 85°C for 2 h at pH 7.0 (Figure 1c) (data for temperature below 85°C were not shown). It retained more than 80% of its activity after standing for 30 min at 90°C, and as indicated the half-life of the recombinant xylanase was approximately 1 h at this temperature (Figure 1c). Furthermore, it was interesting to find that the thermostability of the xylanase benefited from the existence of Ca^2+^ in the solution. The results showed that by adding Ca^2+^ to the final concentration of 5 mM, the xylanase exhibited the highest thermostability, while it was only approximately 20% of its maximum activity without the addition of CaCl_2_. In addition, it was shown that the residual activity enhanced as the final Ca^2+^ concentration increased (Figure 1d). The *K*_*m*_ and *V*_*max*_ of recombinant xylanase for beechwood xylan as the substrate were 2.57 mg mL^-1^ and 325.32 μmol mg^-1^ min^-1^, respectively (Table 1).

**Table 1:** **Characteristics of recombinant xylanase of *T*. *thermarum***

**Property**	**Recombinant Xyn10A**
Specific enzyme activity	145.81 U mg^-1^
Optimum temperature	95°C
Optimum pH	7.0
Thermal stability (55-85°C)	2 h
Molecular weight	131 kDa
*K*_*m*_	2.57 mg mL^-1^
*V*_*max*_	325.32 μmol mg^-1^ min^-1^
*V*_*max*_/ *K*_*m*_	126.58 min^-1^
*k*_*cat*_	710.28 s^-1^
*k*_*ca*t_/*K*_*m*_	276.37 mL mg^-1^ s^-1^

**Figure 2:**
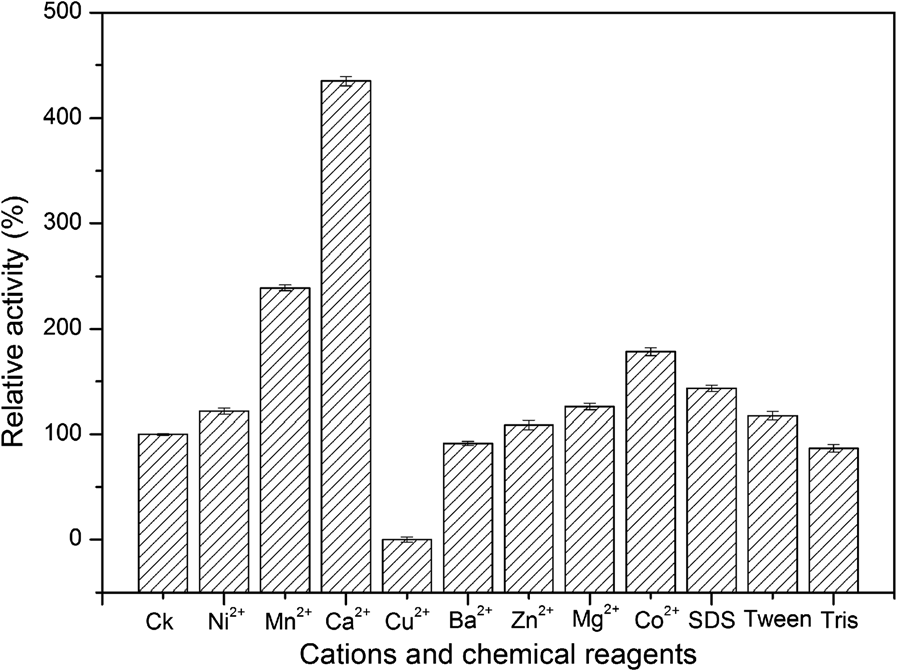
**Effects of cations and reagents on the activity of purified Xyn10A.** The final concentration of cations was 1 mM. The final concentration of reagents detailed as below, 0.05% Tween 60 and Tris, 0.1% SDS. The activity of the experiment without cation or reagent was defined as 100%. Values shown were the mean of duplicate experiments, and the variation about the mean was below 5%.

The effects of metal ions and chemicals on the enzyme activity were also carried out (Figure 2). The results revealed that the enzyme activity was significantly stimulated by Ca^2+^, Mn^2+^, Co^2+^, Al^3+^ and SDS, and was completely inactivated by Cu^2+^. The effects of other metal ions and chemicals on the enzyme activity were insignificant.

### Xylan degradation by Xyn10A

Among the xylans from beechwood, birchwood and oat spelt, the degradation degree of birchwood xylan by xylanase Xyn10A was the highest, followed by beechwood xylan and oat spelt xylan (Table 2, the data on oat spelt xylan was not shown). This result is similar to the xylanase from *Geobacillus Thermoleovorans*[18]. However, xylanase Xyn10A almost had no activity on CMC-Na. After xylans from oat spelt, birchwood and beechwood were hydrolyzed by the recombinant xylanase, the sugars xylose, xylobiose, xylotriose, xylotetraose, xylopentaose and xylohexaose were detected by ICS. Xylobiose, xylotriose and xylotetraose were detected as main products at about 1 h of incubation when birchwood xylan or beechwood xylan was used as substrate. The conversion percentage was about 99% and 89% respectively (Table 2). Nevertheless, when oat spelt xylan was used as substrate, its conversion was only 42% (data not shown).

**Table 2:** Hydrolysis products of birchwood xylan and beechwood xylan

	**0 h**	**0.5 h**	**1 h**	**2 h**	**3 h**	**5 h**
X1	497/533	576/625	677/701	8300/1829	9798/4605	11980/9062
X2	11/44	2200/1870	10203/6938	9674/9094	7962/11362	7494/7955
X3	0/160	5500/4642	7425/7458	1682/5783	1923/1367	209/321
X4	0/152	3200/3298	1017/1278	27/397	0/34	0/60
X5	0/151	2750/2653	334/484	0/52	0/39	0/0
X6	0/0	2820/2532	0/264	0/0	0/0	0/0
Conversion percentage %	2.5/5.2	85.2/78.1	98.3/85.6	98.4/85.8	98.4/87.0	98.4/87.0

X1, X2, X3, X4, X5 and X6 are assigned to xylose, xylobiose, xylotriose, xylotetraose, xylopentaose and xylohexaose respectively. The former value in the table is for birchwood xylan and the latter is for beechwood xylan. The unit is mg L^-1^.

### Phylogeny and catalytic amino acid residues of Xyn10A

**Figure 3:**
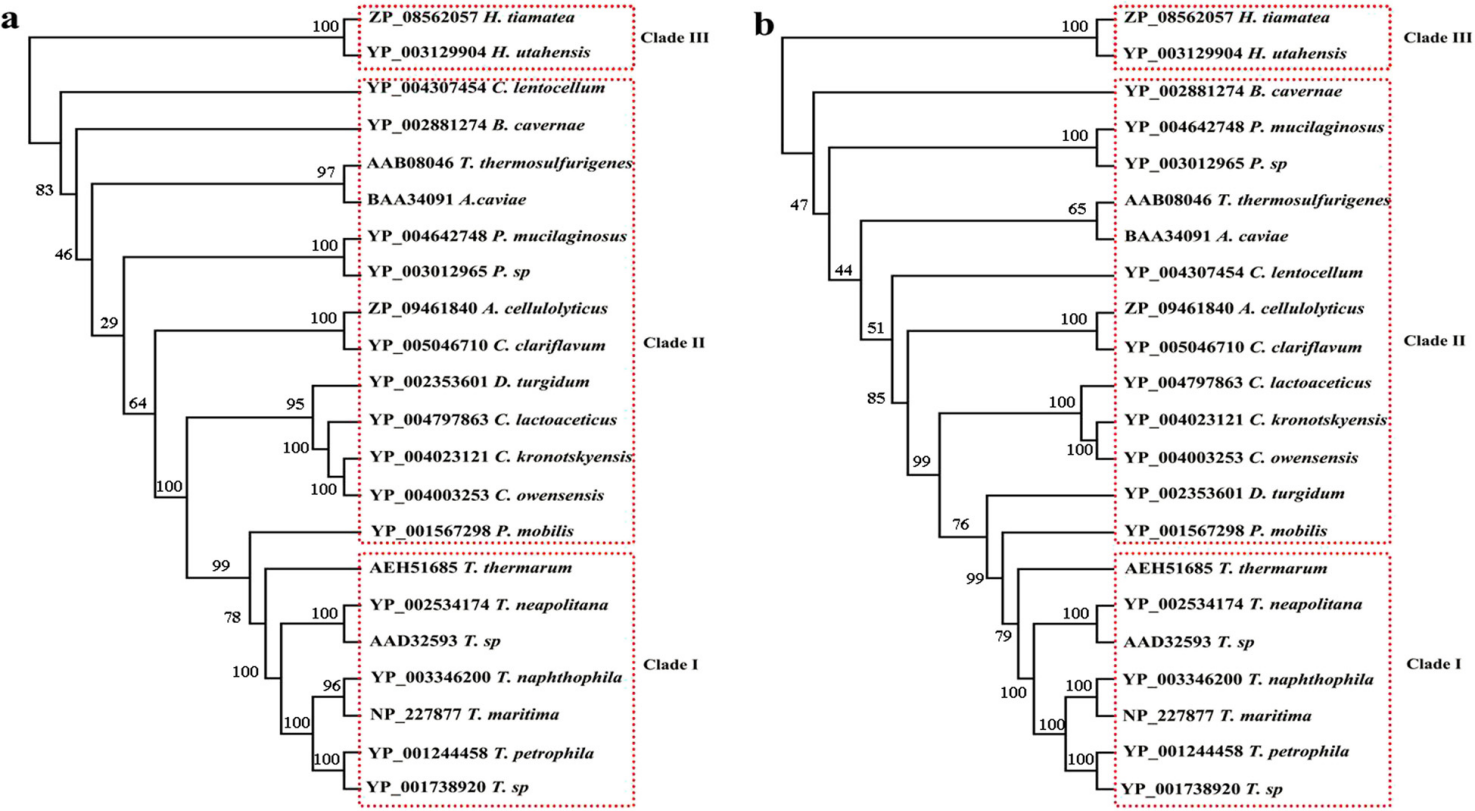
**The Neighbor-Joining (NJ, a) and Maximum-Parsimony (MP, b) trees resulted from analysis of xylanases with 22 amino acid sequences.** Numbers on nodes correspond to percentage bootstrap values for 1000 replicates.

To gain deeper insight into the classification status of Xyn10A among xylanases, the phylogenetic trees generated from 22 candidate amino acid sequences were constructed using the NJ and MP method, which were both supported by similar topology (Figure 3). Three well-supported clades were detected. Clade I was hyperthermophilic xylanases from genus *Thermotoga*, Clade II was xyalnases from other mesothermal and hyperthermophilic bacteria, and Clade III was hyperthermophilic xylanases from archaea. From Figure 3, it was inferred that there existed a monophyletic group in Clade I as Xyn10A xylanase from *T*. *thermarum* (GenBank No: AEH51685), sited in boundary of Clade I, had a good relationship with the xylanase from *P*. *mobilis* in Clade II (GenBank No: YP_001567298).

**Figure 4:**
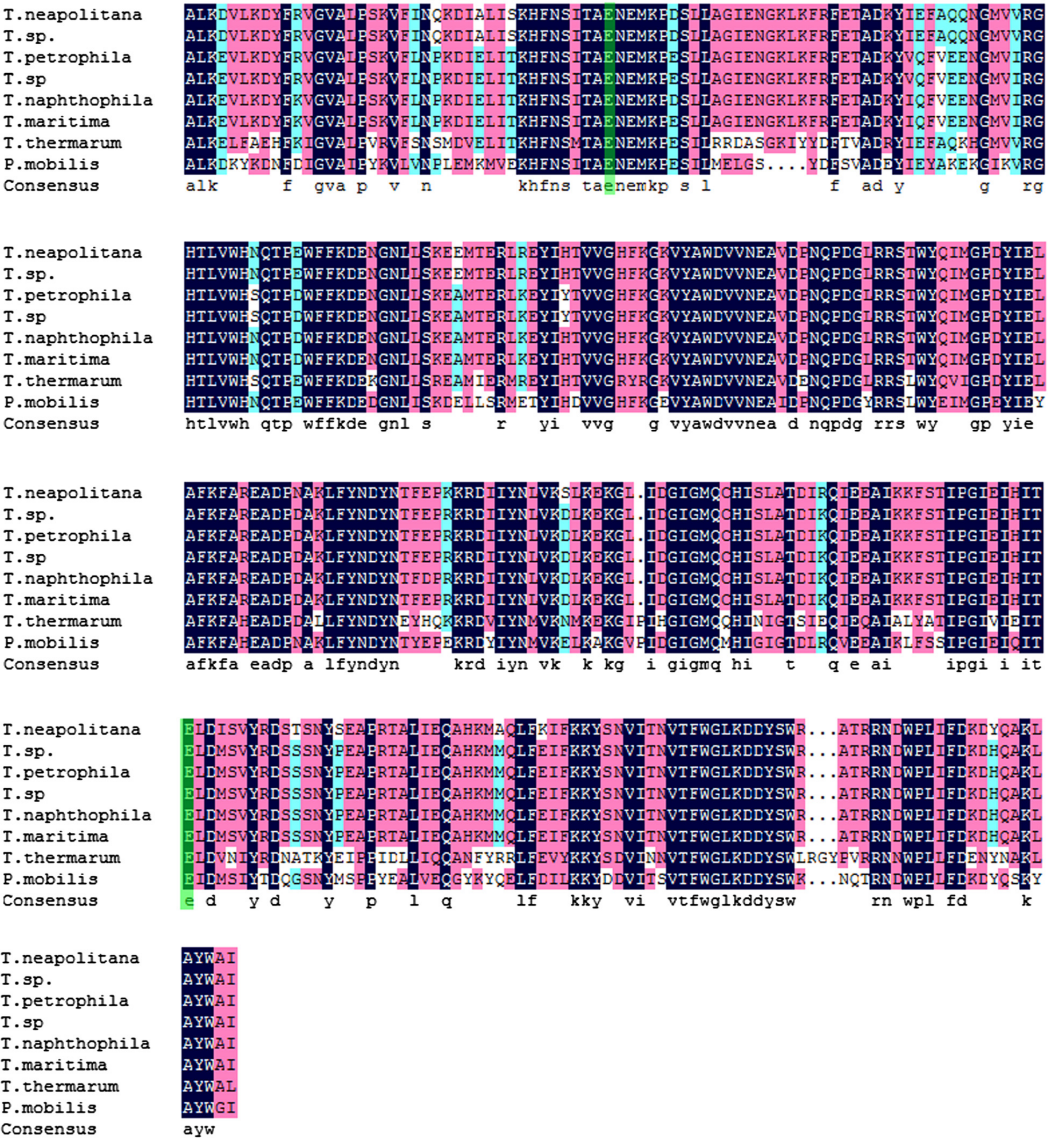
**Multi-alignment of Xyn10A with some GH10 family members.** Sequence alignment was performed by using Clustal X2.0. The active sites were indicated in green. *T*. *neapolitana*: GeneBank No. YP002534174; *T*. *sp*.: GeneBank No. AAD32593; *T*. *petrophila*: GeneBank No. YP001244458; *T*. *sp.*: GeneBank No. YP001738920; *T*. *naphthophila*: GeneBank No. YP003346200; *T*. *maritima*: GeneBank No. NP227877; *T*. *thermarum*: GeneBank No. AEH51685; *P*. *mobilis*: GeneBank No. YP001567298.

Two glutamic acid residues performed as the catalytic nucleophile and proton donor of Xyn10A, which displayed the same function as other xylanases [18,19]. To further investigate the sequence similarity and structural relationship, the amino acid sequences of Clade I and that of the *P*. *mobilis* xylanase were aligned again (Figure 4). The alignments and the predicted three dimensional (3D) structure of catalytic domain (data not shown) showed that Glu525 and Glu726 were conserved among the most diverse members of the GH10 xylanase. This is consistent with the *Thermobifida fusca* Cel9A that three amino acid residues, Asp55, Asp58 and Glu424 are invariant among the majority of GH9 members [20]. Based on sequence alignment and the structures of homologous GH10 xylanases, Glu525 is proton donor while Glu726 is the catalytic base [21]. To further determine which one is the catalytic base or proton donor, an azide rescue test described as Li et al. may be required [22].

## Discussion

Enzymatic hydrolysis of hemicellulose is a complex process, in which several enzymes are required. Among these enzymes, xylanase plays a significant role in the hydrolysis. Members of the genus *Thermotoga* including *T*. *thermarum*, which may produce a number of thermostable enzymes including xylanases, have been isolated from the terrestrial and marine hydrothermal areas. The annotation of genome sequence of *T*. *thermarum* DSM 5069 released recently revealed the presence of a GH10 protein (Lucas S et al., 2011). It shared only 51% sequence similarity with the *Thermotoga petrophila* RKU-1 (Genbank No. YP_001244458) and *Thermotoga sp*. RQ2 (Genbank No. YP_001738920), which both belonged to the genus *Thermotoga*. Compared to other xylanases from the same genus, it has one more carbohydrate binding domain at the C-terminal of the protein through a BLAST search. Furthermore, the GH10 domain of Xyn10A shared only 63% sequence similarity with the *T*. *petrophila* RKU-1 and *T*. *sp*. RQ2. The phylogenetic analysis showed that the Xyn10A and the genus *Thermotoga* xylanases were distant with the hyperthermophilic xylanases from archaea (Figure 3a, b). Hence, it is postulated that a new thermostable xylanase obtained via genetic engineering technology would exhibit some specific properties. Significant amounts of the carboxylate amino acids, Glu and Asp together about 15%, were detected in Xyn10A, which was similar to other heat-tolerant xylanases from the same genus *Thermotoga*[23-26]. From the sequence alignment (Figure 4), it is also indicated that there is almost no Cys detected in those xylanases which is probably critical to their thermostability.

The xylanase Xyn10A from *T*. *thermarum* DSM 5069 exhibited the highest activity at temperature 95°C (Figure 1b). The optimal temperature was higher than the xylanases from *Chaetomium sp*., *Nonomuraea flexuosa*, *Paecilomyces thermophila*, *Talaromyces thermophilus* and *Cellulomonas flavigena*, whose optimal temperature were ranged from 50°C to 80°C [3,7,27,28]. However, all of these thermophilic or hyperthermophilic xylanases share the similar optimal pH near 7.0. Compared to xylanases from the same genus, the xylanase from *Thermotoga neapolitana* exhibited the maximum activity at 102°C [23]. The activity of Xyn10A was completely inhibited by Cu^2+^ and this was similar to the xylanase from *Geobacillu thermoleovorans* which retained approximately 40% of its initial activity upon the addition of 1 mM Cu^2+^[18]. However, the xylanase activity was greatly stimulated by Ca^2+^, Mn^2+^ and Co^2+^, and was enhanced by nearly 400% in the solution of 1 mM Ca^2+^ (Figure 2). It is implied that a few metal ions especially Ca^2+^ is required for maintaining the structure stability, as there are probably two CBMs with Ca^2+^. These results showed that Xyn10A differed from the other bacterial xylanases from *Bacillus subtilis* cho40, *Geobacillus thermoleovorans* and *Paenibacillus campinasensis*, whose activity were not significantly affected by Ca^2+^[2,18,29]. As the longer active life means the less consumption of the enzyme, the high thermostability of the enzyme is desired in industrial production. The Xyn10A residual activity retained nearly 100% after being incubated at 85°C for 2 h with the addition of 5 mM Ca^2+^. Furthermore, the Xyn10A residual activity was still more than 50% even after being incubated at 90°C for 1 h. It exhibited higher thermostability at high temperature above 80°C than thermostable xylanases from *Chaetomium sp*. and *Nonomuraea flexuosa*[3,7]. Sharing similar thermostability from the same genus, *Thermotoga neapolitana* xylanase was stable at 90°C whereas *Thermotoga petrophila* xylanase was only stable at 80°C [23,30].

With efficient conversion of substrate, the *K*_*m*_ and *k*_*cat*_ values of the Xyn10A for beechwood xylan were 2.57 mg mL^-1^ and 710.28 s^-1^ respectively. The *K*_*m*_ value of the enzyme is similar to that of xylanases reported from other bacteria and the same genus bacteria [18,27,30,31]. The specific activities of several commercial thermostable xylanases reported from Sigma-Aldrich (http://www.sigmaaldrich.com) are much lower than Xyn10A. Although the xylanase from *Trichoderma viride* has been reported with activity of 100–300 U mg^-1^ which is higher than Xyn10A, it is not a thermophilic xylanase. The *K*_*m*_ value of the thermostable xylanase from *Thermomyces lanuginosus*, reported 7.3 mg mL^-1^ and 60.2 mg mL^-1^ for soluble and insoluble oat spelt xylan respectively, however, was higher than that of Xyn10A xylanase. Furthermore, the Xyn10A was stable at 85°C for 2h, while *Thermomyces lanuginosus* xylanase was only stable at 60°C for 1 h [32]. Therefore, it can be concluded that Xyn10A exhibits better enzymatic properties for bioconversion of xylan at even higher temperature.

The properties of the xylanase Xyn10A demonstrated a great potential for xylans degradation. The initial hydrolysis products at 0.5 h released from birchwood, beechwood and oat spelt were mainly XOs, and the content of xylose was low (Table 2) (data for oat spelt xylan not shown). This indicated that Xyn10A could be a potential enzyme for the production of XOs. Xylans from birchwood and beechwood both belong to hardwoods. The former are known to be more digestible than the latter due to the chemical modification at side-chains. The xyloses content after hydrolysis were approximately 95% and 90%, respectively. Due to the complex structure and poorer solubility, it was hard to hydrolyze oat spelt xylans. The content of arabinose, glucose and xylose were approximately 10%, 15% and 70%, respectively. Xyloses, clearly detected in the end products, together with xylobioses were the major products of birchwood and beechwood xylan degradated by the recombinant enzyme Xyn10A after 5 h hydrolyzation (Table 2). This is in accordance with that of GH10 xylanase from *Thermoascus aurantiacus* in which the xyloses and xylobioses are the main end products after prolonged hydrolysis for 48 h [7]. Therefore, the desired end products can be simply and conveniently obtained according to the hydrolysis time. The feature will enable Xyn10A as an effective and powerful enzyme for large scale production of XOs which can be used as prebiotics for fish breeding and poultry raising [33].

## Conclusions

In this study, a novel β-xylanase, Xyn10A from *T*. *thermarum* DSM 5069 was obtained with a few specific features. The phylogenic analysis showed that the Xyn10A had close relationship with the β-xylanases from hyperthermophilic bacteria. Compared with the properties of enzyme from other microorganisms, the Xyn10A with higher thermostability is more efficient in xylan hydrolysis. Therefore, this study will provide a novel and effective β-xylanase with combined properties of high thermostability and broad thermal and pH stability. It is easily envisioned that the recombinant β-xylanase can be used to improve the enzymatic conversion of hemicellulose to xylooligosaccharides or xyloses through synergetic action.

## Materials and methods

### *xyn10A* gene, plasmid, growth media

The xylanase gene *xyn10A* from *Thermotoga thermarum* was synthesized by Genray Biotech Co., Ltd (Shanghai China). *Escherichia coli* BL21 (DE3) was grown at 37°C in Luria-Bertani medium (LB) and supplemented with ampicillin when required. The expression vector pET-20b (Novagen) was employed as cloning and expression vector.

### DNA manipulation, plasmid constructions

DNA was manipulated using standard procedures. Biomiga Plasmid Kit and Biomiga Gel Extraction Kit (Biomiga, China) were used for the purification of plasmids and PCR products. DNA restriction and modification enzymes were purchased form TaKaRa (Dalian, China). DNA transformation was performed by electroporation using GenePulser (Bio-Rad, USA).

The xylanase gene *xyn10A* was amplified from synthesized sequences by PCR using primers *Tth*-1 and *Tth*-2 as follows, *Tth*-1 5^′^-GGAATTCCATATGGCAGTTGTGGCAAACTACGATTTTGAGAC-3^′^, *Tth*-2 5^′^-CCGCTCGAGCTTAGTCAGGATCAGGTTG-3^′^. The PCR products were then digested with *NdeI* and *XhoI* and inserted into pET-20b at the corresponding sites, yielding the plasmid pET-20b-*xyn10A*.

### Expression and purification of the recombinant xylanase Xyn10A

Plasmid pET-20b-*xyn10A* was transformed into *E*. *coli* BL21 (DE3), and induced to express recombinant Xyn10A by adding isopropyl-β-D-thiogalactopyranoside (IPTG) to final concentration of 0.5 mM at OD_600_ approximately 0.8, and incubated further at 30°C for 12 h.

200 mL of the recombinant cells carrying Xyn10A were harvested by centrifugation at 10,000 g for 15 min at 4°C, washed twice with 20 mM Tris-HCl buffer (pH 8.0), and re-suspended in 5 mL of 5 mM imidazole, 0.5 M NaCl, and 20 mM Tris-HCl buffer (pH 7.9). The cell extracts after sonication were heat treated at 70°C for 30 min, cooled in an ice bath, and then centrifuged (15,000 g, 4°C, 20 min). The resulting supernatants were loaded onto an immobilized metal affinity column (Novagen, USA), and eluded with 0.4 M imidazole, 0.5 M NaCl, and 20 mM Tris-HCl buffer (pH 7.9). SDS-PAGE was carried out to verify the purity of the target proteins [34], and the protein bands were analyzed using an image analysis system (Bio-Rad, USA). The concentration of the purified protein was determined by the Bradford method using BSA as a standard.

### Determination of enzyme activities and properties

Beechwood xylan, birchwood xylan and oat spelt xylan were purchased from Sigma-Aldrich (St. Louis, USA). The reaction mixture, containing 50 mM imidole-potassium buffer (pH 6.5), 0.5% xylan from beechwood, and a certain amount of xylanase in 0.2 mL, was incubated for 10 min at 85°C. The reaction was stopped by the addition of 0.3 mL 3,5-dinitrosalicylic acid (DNS) [35], followed by boiling for 5 min. The absorbance of the mixture was measured at 550 nm and converted to micromole of xylose by a xylose standard curve (data not shown). One unit of enzyme activity was defined as the amount of enzyme necessary to liberate 1 μmol of reducing sugars per min under the assay conditions. All the values of enzymatic activities shown in figures were averaged from three replicates.

The optimum pH for xylanase was determined by incubation at various pH conditions (pH 4.0 - 8.5) at 85°C for 10 min in 50 mM imidole-potassium buffer. The optimum temperature for the enzyme activity was determined by standard assay ranging from 55°C to 100°C in 50 mM imidole-potassium buffer at pH 6.5. The results were expressed as relative activity to the value obtained at either optimum temperature assay or optimum pH assay.

The pH stability of the enzyme was determined by measuring the remaining activity after incubating the enzyme (0.01 μg) at 90°C for 2 h in 50 mM imidole-potassium buffer from pH 4.0 to 8.5. To determine the effect of temperature on the stability of xylanase Xyn10A, the enzyme (0.01 μg) in the presence of 5 mM Ca^2+^ in 50 mM imidole-potassium buffer (pH 7.0) was pre-incubated for various time (0 min, 30 min, 60 min, 90 min, 120 min) at 85°C, 90°C and 95°C in the absence of the substrate. The activity of the enzyme without pre-incubation was defined as 100%.

Kinetic constant of xylanase Xyn10A was determined by measuring the initial rates at various beechwood xylan concentrations under standard reaction conditions. The *K*_*m*_ and *V*_*max*_ were determined at 85°C by recording the effect of various concentrations (0.05%, 0.1%, 0.15%, 0.2%, 2.5%, 3%) of beechwood xylan.

The effects of metal ions and chemical reagents on enzyme activity of purified xylanase (0.01 μg) were determined by applying Mg^2+^, Zn^2+^, Ba^2+^, Ni^2+^, Mn^2+^, Ca^2+^, Co^2+^, Cu^2+^ and Al^3+^ at a concentration of 1 mM, and the chemical reagents Tween 60, Tris and SDS at 0.05%, 0.05% and 0.1%, respectively in the reaction mixture. The enzyme was incubated with each reagent for 2 h at 85°C before the addition of xylan to initiate the enzyme reaction. The activity of the enzyme without adding chemical reagents or metal ions was defined as 100%.

### Analysis of hydrolysis products

The enzymatic production of xylose and xylooligosaccharides was carried out at 85°C for different time, and the hydrolysis products were analyzed using ICS (Dionex, USA). 4 mg of xylan (from oat spelt, birchwood or beechwood) was incubated with 1 μg of recombinant xylanase in 200 μL 50 mM imidole-potassium buffer (pH 7.0) at 85°C for 0.5 h, 1 h, 2 h and 3 h. The samples were then collected at different time intervals and the resulting sugars were analyzed by ICS. The sugars were identified by comparing the diluting time of the standards of xylose, xylobiose, xylotriose, xylotetraose, xylopentaose and xylohexaose (Sigma, USA).

### Multiple sequence alignment, phylogenetic analysis

Amino acid sequence of hyperthermophilic xylanase Xyn10A (GenBank No: AEH51685) was used as a BLAST query for seeking other homologous amino acid sequences. In this study, the sequence alignment was created with Clustal X2 program [36]. Sequences were further edited and aligned manually using the Mega 5 software when necessary [37]. Phylogenetic relationship was inferred using the Neighbor-Joining (NJ) and Maximum-Parsimony (MP) method as implemented in Paup 4.0 [38]. The NJ and MP trees, displayed using TREEVIEW 1.6.6 (http://taxonomy.zoology.gla.ac.uk/rod/treeview.html), were evaluated with 1000 bootstrap replicates.

### Three-dimensional structure prediction

For homology modeling, the crystal structure of the xylanase (EXPDB ID: 1n82A) was used as a template. The 3D structure of catalytic domain (CD) of the Xyn10A was obtained [39-41] and further modified by PyMOL (version 1.4.1, http://www.pymol.org/).

## Abbreviations

GH: Glycoside hydrolase;ICS: Ion Chromatography System;ORF: Open reading frame;IPTG: Isopropyl-β-D-thiogalactopyranoside;SDS: Sodium dodecyl sulfate;SDS-PAGE: SDS-polyacrylamide gel electrophoresis;CMC-Na: Sodium carboxymethyl cellulose;NJ: Neighbor-Joining;MP: Maximum-Parsimony;Asp: Aspartic acid;Glu: Glutamic acid;CBM: Carbohydrate binding module;XOs: Xylooligosaccharides;NaCl: Sodium chloride;CaCl2: Calcium chloride;BSA: Bovine serum albumin

## Competing interests

The authors declare that they have no competing interests.

## Authors’ contributions

HS carried out the cloning and expression and drafted the manuscript. YZ helped to analyze the degradation products and revise the manuscript. XL, LW, YW, HD and YH helped to purify and characterize the Xyn10A. FW directed the over-all study and revised the manuscript. All authors have read and approved the final manuscript.
